# Differential Immune Response to Infection and Acute Inflammation Along the Epididymis

**DOI:** 10.3389/fimmu.2020.599594

**Published:** 2020-11-27

**Authors:** Christiane Pleuger, Erick José Ramo Silva, Adrian Pilatz, Sudhanshu Bhushan, Andreas Meinhardt

**Affiliations:** ^1^Institute of Anatomy and Cell Biology, Justus-Liebig-University Giessen, Giessen, Germany; ^2^Hessian Centre of Reproductive Medicine, Justus-Liebig-University Giessen, Giessen, Germany; ^3^Department of Biophysics and Pharmacology, Institute of Biosciences of Botucatu, São Paulo State University (UNESP), Botucatu, Brazil; ^4^Department of Urology, Pediatric Urology and Andrology, University Hospital, Justus-Liebig-University Giessen, Giessen, Germany

**Keywords:** epididymis, epididymitis, mononuclear phagocytes, uropathogenic *E. coli*, infertility, bacterial infection

## Abstract

The epididymis is a tubular structure connecting the vas deferens to the testis. This organ consists of three main regions—caput, corpus, and cauda—that face opposing immunological tasks. A means of combating invading pathogens is required in the distally located cauda, where there is a risk of ascending bacterial infections originating from the urethra. Meanwhile, immune tolerance is necessary at the caput, where spermatozoa with immunogenic neo-antigens originate from the testis. Consistently, when challenged with live bacteria or inflammatory stimuli, the cauda elicits a much stronger immune response and inflammatory-inflicted damage than the caput. At the cellular level, a role for diverse and strategically positioned mononuclear phagocytes is emerging. At the mechanistic level, differential expression of immunoprotective and immunomodulatory mediators has been detected between the three main regions of the epididymis. In this review, we summarize the current state of knowledge about region-specific immunological characteristics and unveil possible underlying mechanisms on cellular and molecular levels. Improved understanding of the different immunological microenvironments is the basis for an improved therapy and counseling of patients with epididymal infections.

## Structure and Function of the Epididymis

**Figure 1:**
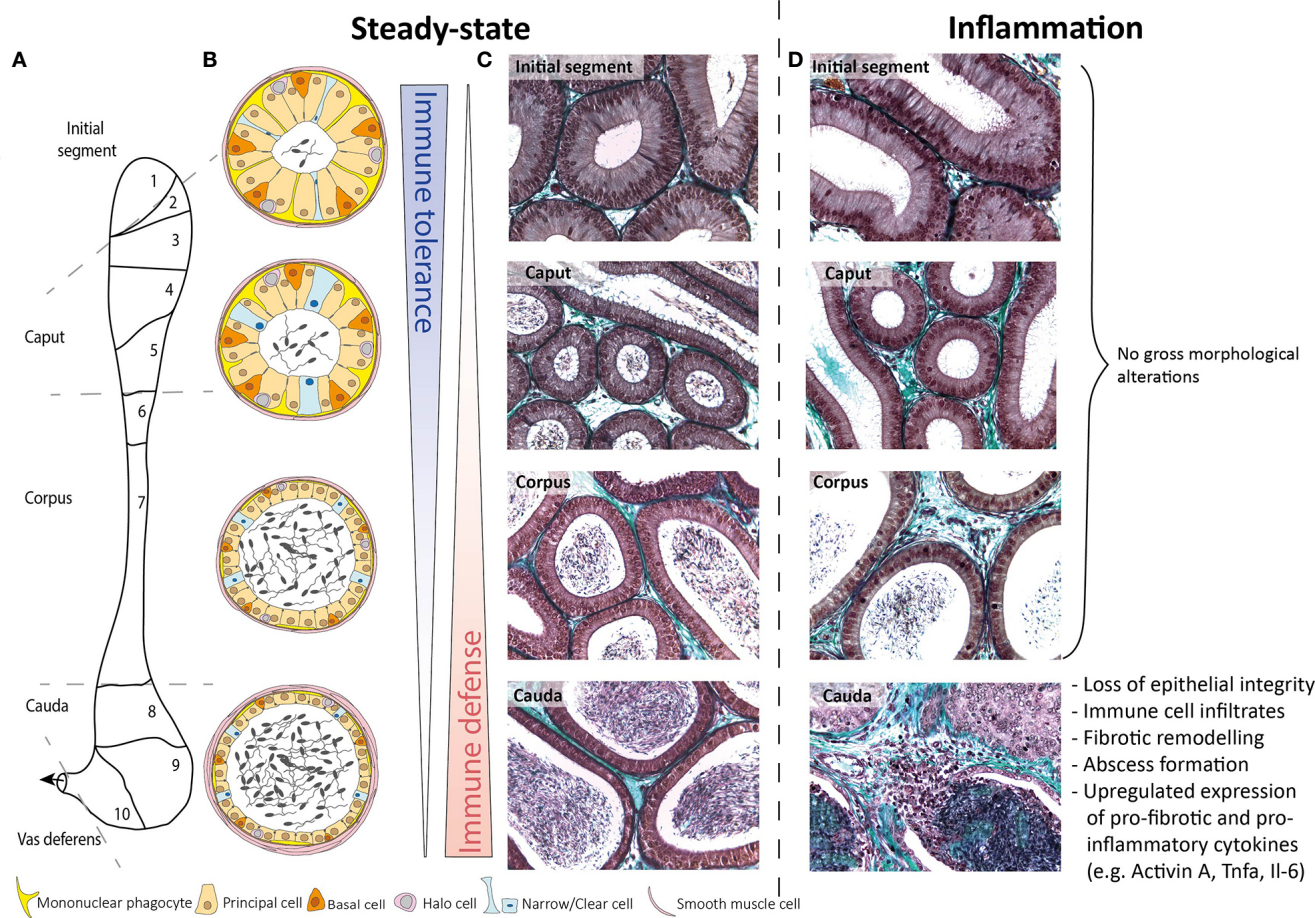
Schematic overview of murine epididymal regions during steady-state and inflammatory conditions. **(A)** Based on structure and function, the murine epididymis is principally compartmentalized into four distinct anatomical regions, i.e. the initial segment (segment 1), caput (segments 2–5), corpus (segments 6–7), and cauda (segments 8–10). **(B)** The epididymal epithelium consists of diverse cell types, namely principal cells, basal cells, halo cells, and clear/narrow cells, whereby the epithelial composition and lumen diameter changes along the length of the single duct. To fulfill the main immunological functions—immune tolerance in the proximal regions and immune defense in the distal regions—mononuclear phagocytes are strategically positioned between adjacent epithelial cells and exhibit long protrusions within the initial segment, which are gradually shrinking towards the distal regions. **(C, D)** Under inflammatory conditions (10 days after infection with uropathogenic *E. coli*), the epididymis shows striking different immunological responses. While the initial segment, caput, and corpus remain mostly unaffected, the cauda epididymidis undergoes dramatic morphological alterations. Masson-Goldner-Trichrome staining, primary magnification 40×.

The epididymis is connected to the rete testis *via* efferent ducts that converge onto one single epididymal duct in the initial segment opposite the mediastinum testis. The epididymal duct meanders through three regions known as the caput (which contains the initial segment in rodents), corpus, and cauda epididymides (**Figure 1A**). Connective tissue septae segregate the epididymis further, producing 10 segments in mice (1) and 19 in rats (2). Septae in the human epididymis are present but poorly defined (3). The duct is lined by a two-layered pseudostratified epithelium, consisting of principal, basal, narrow/clear cells, and resident immune cells [mononuclear phagocytes (see *Immune-Cell Populations in the Rodent Epididymis*) and “halo cells” (4, 5)] and surrounded by a wall composed mostly of smooth muscle cells (**Figures 1B, C**). The interstitium is composed of blood vessels, lymphatic vessels, leukocytes, and loose connective tissue, which increases in density in the septae. The luminal fluid provides a milieu necessary for the step-wise maturation of spermatozoa required to achieve full fertilizing capacity (6, 7). The blood-epididymal-barrier (BEB)—extending apically between adjacent principal cells—maintains the unique composition of the luminal fluid. However, its role in protecting spermatozoa from immune attack is likely not as robust as the blood-testis-barrier (8).

Emerging evidence on cellular and molecular levels has resulted in the hypothesis that the epididymis is “a series of organs side-by-side” (9). This hypothesis is evidenced by particular region-specific characteristics in regards to (a) the composition of the epithelium (4, 5), (b) the distribution and phenotype of resident immune cells subpopulations (10–13), and (c) differential gene expression profiles (1, 2, 14, 15). In line with that, septae segregate different segments and have been demonstrated to function as diffusion barriers, possibly creating distinct interstitial microenvironments (16, 17).

From the immunological perspective of the hypothesis that the epididymis is a “series of organs side-by-side,” this duct faces two different immunological challenges at its opposing ends (**Figure 1B**). At the proximal end, mechanisms of strong local tolerance are required to avoid autoimmune reactions against post-testicular spermatozoa expressing sperm-specific neo-antigens (18). Conversely, at the distal end, mechanisms are required to react against bacterial pathogens ascending from the urethra (19). As pointed out below, there is accumulating evidence that different subsets of resident immune cell populations exist throughout the length of the epididymis that may play a role in the balance of immune tolerance and immune defense at the opposing ends of this organ.

## Leukocytes in the Normal Epididymis

### Immune-Cell Populations in the Rodent Epididymis

Different immune-cell populations, including heterogenous subsets of the mononuclear phagocyte (MP) system, T and B lymphocytes reside within the rodent epididymis (4, 10, 11, 13, 18, 20–23). Two different T-cell populations [CD4^-^CD8^-^ (DN) and γδ T cells] have been identified recently: DN T cells are more abundant within the caput while γδ T cells are evenly distributed throughout the entire epithelium and interstitium (11). A region-specific role for these subpopulations remains elusive.

Generally, tissue-resident MPs are considered “guardians of the immune system,” often found located at the interface with the external environment. MPs comprise heterogeneous subsets of monocytes, macrophages, and dendritic cells (DCs), which have a broad spectrum of common characteristics (i.e. migratory capabilities towards gradients of microbial signals or chemokines, engulfment and processing of microbial fragments or dying cells and antigen-presentation to the adaptive immune system, secretion of signaling molecules). Most notably, MP subsets are highly plastic, allowing them to regulate tissue homeostasis and immune responses in an organ-specific manner.

Within the epididymis, MPs constitute the majority of resident immune cells and comprise multiple closely related subpopulations that are strategically positioned throughout the different regions. As the composition and phenotype of epididymal MPs under normal circumstances has been comprehensively summarized elsewhere (24, 25), only a brief reflection will be provided here serving as a prerequisite to understand alterations seen in infection and inflammation. In brief, distinct MP populations at the periphery of the epithelium and within the interstitium are arranged as dense network with the highest abundance in the initial segment (IS). They are characterized by the expression of the chemokine (C-X3-C motif) receptor 1 (CX_3_CR_1_) as well as a region-specific morphology and transcriptomic signature (10, 13, 24). While peritubular CX_3_CR_1_^+^ cells within the IS exhibit long arborizations between adjacent epithelial cells towards the tight junctions of the BEB, these gradually decline towards the distal segments until the cells finally appear flat in the cauda [**Figure 1B** (13)]. Interstitial MP do not exhibit a stellate morphology. Rather, they can be differentiated by their expression of the macrophage mannose receptor CD206 and lack of CD11c, pointing to a macrophage phenotype (13).

Epididymal MPs are currently classified into different subtypes based on the expression of surface markers traditionally used to distinguish macrophages from DCs (10, 13), i.e. the high-affinity IgG receptor FcγR CD64 *vs.* integrin alpha X CD11c expression, respectively (26, 27). However, several subsets have been described to express a complex combination of these markers in association with other markers (CX_3_CR_1_, CD11b, F4/80) (10). Thus, the original classification for epididymal MPs requires further consideration, with the benefit of techniques such as single cell RNA-sequencing and lineage tracing, to ultimately clarify the full extent of MP heterogeneity. Indeed, a recently published transcriptomic analysis revealed distinct gene expression profiles of CX_3_CR_1_^+^ MPs isolated from different epididymal regions (10) suggesting a considerable level of MP heterogeneity and microenvironment-specific functions beside archetypical functions in all regions (i.e. phagocytosis and antigen-presentation). As an example, CX_3_CR_1_^+^ MPs within the IS are differentially enriched with transcripts required for leukocyte transendothelial migration and cell adhesion (see below). Meanwhile, CX_3_CR_1_^+^ MPs within the cauda are characterized by transcripts associated with NF-kappa B signaling, indicative of their protective role against invading luminal pathogens (10). Intriguingly, while both tubule-associated and interstitial MPs within the highly vascularized proximal regions (in particular the IS) can capture and process circulatory antigens, only interstitial MPs exhibit these properties in the more scarcely vascularized cauda (10). These spatial differences prompt the hypothesis that in the caput intraepithelial MPs could be involved in the continued capture of sperm autoantigens to maintain tolerance, while both the interstitially located MPs in the caput and cauda shall restrict microbial dissemination into the organ.

### Immune-Cell Populations in the Human Epididymis

In contrast to experimental animals, human data on the epididymal leukocyte population are scarce and are almost exclusively derived using immune-based morphological methods (28). Human intraepithelial lymphocytes and macrophages are similar in terms of location to those found in rodents (29). Macrophages are the predominant immune cell population—except for intraepithelial leukocytes where T lymphocytes exceed—and express major histocompatibility complex class II (MHC-II) proteins in the interstitium. B cells are barely detectable, while DC consist of immature DC in the normal epididymis [CD1a^+^ DC, CD11c^+^ myeloid DC (mDC), and CD209^+^ DC] (30, 31). Plasmacytoid DC and CD83^+^ mature DC are only found in chronic epididymitis, similar to CD4^+^Th17^+^ T lymphocytes (30). Although not entirely clear, MHCII-restricted CD4^+^ T lymphocytes seem to represent the predominant phenotype of T cells in the interstitium. As in other organs, CD8^+^ T cells are the predominant phenotype in the epithelium and increase distally in the epididymis (31–33).

## The Epididymal Response to Infection and Inflammation

Various *in vivo* and *in vitro* models have clearly pointed to striking region-specific differences in the epididymal immune response. The clearest observation is that the cauda epididymidis is much more sensitive to inflammatory-inflicted damage than the caput epididymidis [**Figure 1D** (14, 34, 35)]. These observations are complemented by data from patients with acute bacterial epididymitis where in most cases the cauda is predominantly impacted, particularly in severe cases when an abscess is diagnosed (36).

### Region-Specific Responses in Human Epididymitis

Acute epididymitis affects ~250 to 650 per 100,000 men each year (37). Three different forms can be distinguished: i) bacterial ascension through the male accessory glands (the most common form), ii) concomitant epididymitis in the context of viral orchitis, and iii) a primarily viral epididymitis.

**Figure 2:**
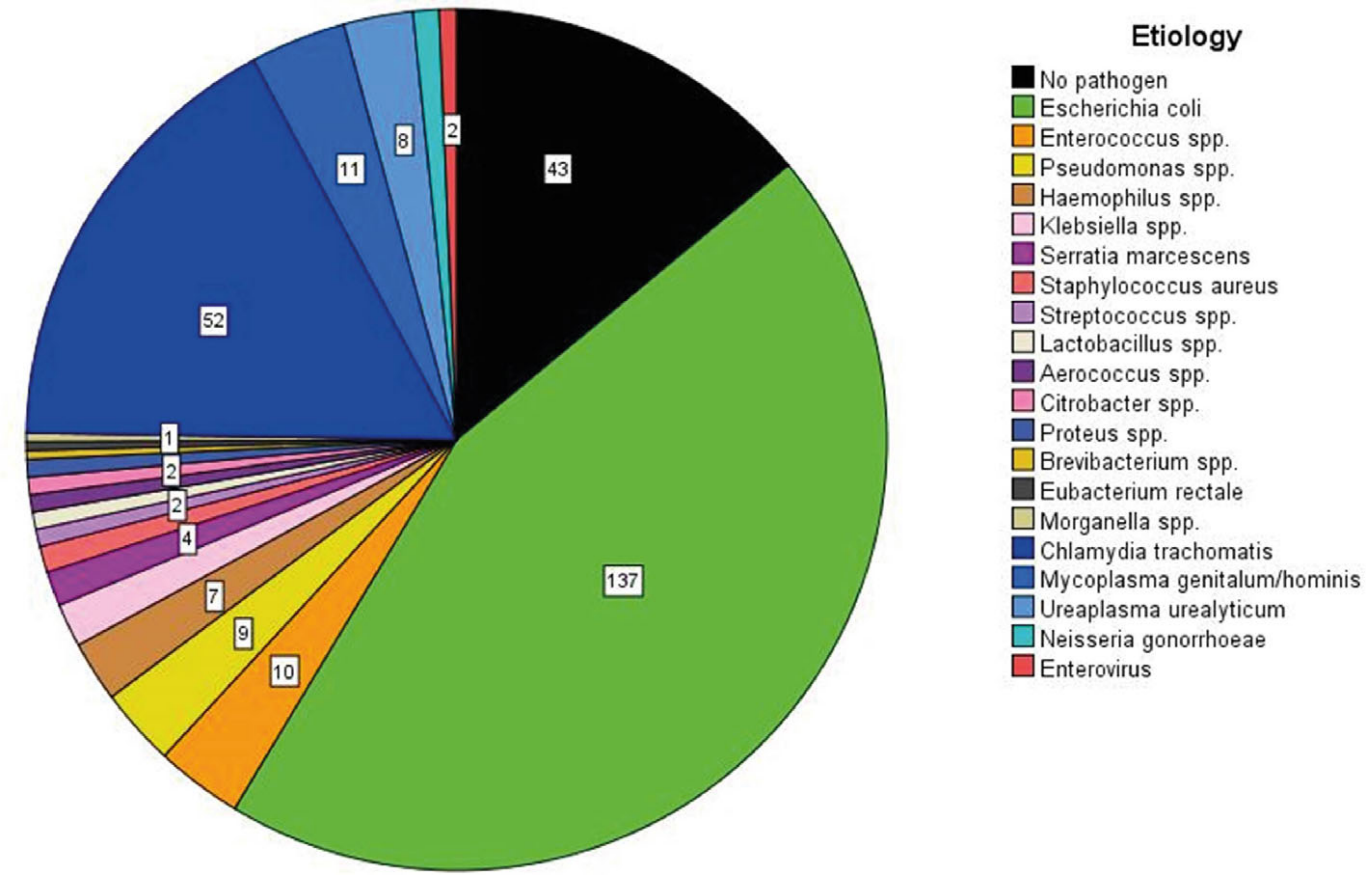
The etiology of acute epididymitis in 284 patients without antimicrobial pre-treatment. A total of 307 pathogens are visualized due to two specimens being simultaneously detected in 23 patients. The pathogen detection rate is 85% with *E. coli* as the most common pathogen, followed by *Chlamydia trachomatis* as second most common. Data are obtained from a prospective study running since 2007 [modified according to (36)].

Molecular microbiological diagnostics can identify a causal bacterial pathogen in 87% of antibiotic-naïve cases (36). Both typical urinary tract pathogens [*Escherichia coli* (*E. coli*)] and sexually transmitted bacteria (*Chlamydia trachomatis, Neisseria gonorrhoeae*) are most relevant, regardless of age [**Figure 2** (36)]. As a result of bacterial ascension, 44% of patients experience isolated pain in the cauda epididymidis, 41% experience pain in the entire epididymis, and only 15% report isolated pain in the caput epididymidis (36). Abscess formation characterizes clinically severe forms of the disease. Interestingly, abscesses are typically located in the cauda, and very rarely in the caput epididymidis (36). Early studies that collected diagnostic epididymal biopsies identified polymorphonuclear cells in ~54% of cases and somewhat less frequently lymphocytic infiltrates (38). Of note, no differentiation of the inflammatory response in dependence of the etiology of the most relevant pathogens (e.g. *E. coli* vs. *Chlamydia trachomatis*) was ever conducted in human specimens. Research of this kind is limited by the low availability of specimens as histological evaluation is only performed in cases with a fulminant course, where secondary testicular infarction with the necessity of semi-castration occurs (36). Finally, persistent epididymal enlargement (usually of the cauda) >3 months post infection affects ~16% of patients (36).

Concomitant epididymitis in primary viral orchitis is possible both synchronously and metachronically, but the frequency is unclear. Data only exist for mumps orchitis from the era before mumps vaccination, and when scrotal ultrasound was unavailable (39). Currently, only one study has used ultrasound to describe the involvement of the epididymis in 23 cases of mumps orchitis. Interestingly, in 45% of the cases, no epididymal changes were visible (isolated orchitis), while in 30% only the caput was affected (diameter 11.1 mm instead of 6.5 mm) and in another 26% the entire epididymis was affected (40). These findings are in stark contrast to those of bacterial epididymitis, where the cauda is always primarily affected.

Only few studies carried out virological examinations in epididymitis patients. In a study investigating 28 epididymal biopsies, arbor and rhinoviruses were detected in only three cases (38); unfortunately corresponding histological reports were lacking. With epididymal biopsies now obsolete due to the risk of obstruction, another study isolated enteroviruses directly from the semen of patients with acute epididymitis and considered this as the etiologic pathogen (36). Moving forward, PCR-based urine diagnostics (STIs and 16s rDNA) are now recommended in the case of antibiotic pre-treatment with negative urine culture, while PCR diagnostics should be performed directly from the ejaculate if viral genesis is suspected (36).

### Region-Specific Immune Responses in Rodent Models of Acute Bacterial Epididymitis

A frequently used rodent model to mimic ascending acute bacterial epididymitis uses uni- or bilateral intravasal inoculation of uropathogenic *E. coli* [UPEC, (19)]. After infection, fundamentally disparate immune responses and associated immunopathologies were observed in different epididymal regions (**Figures 1C, D**). The caput epididymidis remains mostly unaffected throughout the course of infection despite the presence of bacteria and the expression of signaling molecules required to sense bacteria and mount inflammatory responses against them [see *Region-Specific Immune Responses to LPS-Induced Epididymitis* (14, 34)]. Similar region-specific immune reactions have also been observed in a mouse model of experimental autoimmune epididymo-orchitis (35).

#### Immunotolerance in the Caput Epididymidis

A possible explanation for different immune responsiveness could lie in the unique requirements of the immune environment of the caput. Here, neoantigen-expressing spermatozoa originate from the immune-privileged testicular environment. As a result, multiple complementary mechanisms of peripheral immune tolerance are established to prevent autoimmune reactions against spermatozoa. At the cellular level, intraepithelial MPs sample sperm neoantigens *via* their characteristic thin protrusions (see *Immune-Cell Populations in the Rodent Epididymis*) that reach the epididymal lumen and—after migration to draining lymph nodes—induce regulatory T cells to suppress effector T cells (41). Although this mechanism was observed in men after vasectomy and may not reflect the steady-state situation, support for a more general mechanism in epididymal function has been obtained in the gut, an organ that faces similar challenges in inducing peripheral tolerance. In the gut, CX_3_CR_1_^+^ macrophages (located within the lamina propria) capture luminal antigens and transfer them to CD103^+^ DC in a connexin43-dependent manner (42). CD103^+^ DC, in turn, migrate through lymphatics to the draining lymph node to induce the differentiation of antigen-specific regulatory T cells (42, 43).

In addition, MPs residing in the IS are in close contact with neighboring epithelial cells and possess a strong capacity to resolve arising inflammation—as shown by the rapid clearance of damaged epithelia (44). These and other means to preserve epithelial integrity are important mechanisms to maintain peripheral tolerance as a loss of epithelial integrity in the IS promotes autoimmune-associated responses against sperm antigens as seen in mice lacking the adhesive protein milk fat globule-EGF factor 8 protein MFGE8 (also known as SED1 or lactadherin) (45). Moreover, an important role for regulatory T cells was postulated in epididymal immune tolerance as depletion of these cells allowed induction of experimental auto immune-orchitis, at least under certain conditions (vasectomized B6AF1 mice) (46).

Molecular data point to the necessity to preserve immunotolerance not only in the IS and caput, where sperm first enter the epididymis, but throughout the organ. Here, it seems that transforming growth factor-beta (TGFβ) family members have a prominent role. TGFβ isoforms (TGF-β1, TGF-β2, TGF-β3) are expressed in all epididymal regions, but the active forms are preferentially found in the corpus (47). All isoforms activate the same receptor complex (TGFβ receptor 1 and 2) (48). Mice with DC-specific TGFβ receptor 2 deficiency develop severe leukocytosis in all regions, including the IS, which is triggered by an immune response specifically targeting sperm antigens (18).

Other immunomodulatory molecules, such as indolamine 2,3-dioxygenase [IDO (49)] or activin (50) are mostly enriched in the proximal region. Further protective mechanism in the proximal region of the epididymis could be based on the high expression of a large number of various anti-microbial peptides (51, 52). These include beta-defensins (*Defb* 3, 12, 15, 18, 20, 30, 42), lipocalin 2 (*Lcn2*), cathelicidin (*Camp*), pentraxin 3 (*Ptx3*), and *Lypd8* (Ly6/Plaur domain -containing protein 8). All of them were found in the top 50 significantly differential expressed genes with high baseline expression in the normal caput epididymis using whole transcriptome analysis (14). While beta-defensins are mostly expressed by epithelial cells, including proton-secreting clear cells with protrusions reaching into the lumen (53) for other anti-microbial peptides their cellular origin in the epididymis is not established, e.g. those controlling infection with gram-negative bacteria (*Lcn2*, *Lypd8*). It needs to be noted though that other defensins such as *Defb* 1, 2, and 9 are expressed at much higher levels in the cauda. This indicates a complex multitude of mechanisms that contribute to the maintenance of peripheral tolerance, whilst simultaneously providing sufficient resistance to bacteria-induced damage [reviewed in (54)], it is not clear to what extend their differential distribution contributes to immune tolerance and a dampened immune response towards microbes preferentially in the proximal epididymis.

#### Immune Defense in the Cauda Epididymidis

Conversely, the cauda is highly susceptible to damage as a result of inflammatory responses. UPEC-infected mice are characterized by interstitial and intraluminal immune cell infiltrates, epithelial detachment, and fibrosis that all contribute to irreversible epididymal duct obstruction [**Figure 1D** (14, 34)]. The immunopathological damage gradually decreases towards the corpus epididymidis. Besides the transcriptional differences underlying the immune responsiveness of epididymal MPs (10), segmental barriers serve to restrict the ascent of pathogens and spread of inflammatory modulators, at least for some time (16).

Intriguingly, the observed damage is mostly elicited by the magnitude of the host’s immune response and only to a lesser extend due to bacterial virulence factors as evidenced by mice lacking the Myeloid Differentiation Primary Response Protein MYD88, which are characterized by a less pronounced immune reaction towards UPEC infection (34).

Although antibiotic interventions can eliminate epididymitis-associated pathogens, histopathological alterations and concomitant long-term fertility impairments are not preventable by antibiotics alone (55–59). However, a combined antibiotic (i.e. levofloxacin) and immunosuppressant (dexamethasone) regimen can successfully dampen adaptive (rather than innate) immune responses and the associated tissue damage in UPEC-induced mouse epididymitis (59). This treatment might thus constitute a promising strategy for fertility preservation in affected men (59).

### Region-Specific Immune Responses to LPS-Induced Epididymitis

Considering the frequency of *E. coli*-induced epididymitis in men (see *Region-Specific Responses in Human Epididymitis*), lipopolysaccharide (LPS), a structural component of the outer membrane of Gram-negative bacteria such as *E. coli*, can be explored as agonist of the toll-like receptor 4 (TLR4) to investigate the role of TLR4 signaling in epididymitis pathogenesis. To study region-specific immune responses, LPS can be applied systemically to circumvent the temporally different exposures of the epididymal regions to the inflammatory challenge, as seen in models of ascending bacterial infection (14, 34). In rodents, TLR4 is expressed in epididymal epithelial cells, smooth muscle cells and interstitial macrophages (60–63). TLR4 and associated signaling molecules [i.e. MYD88, cluster differentiation 14 (CD14), TIR-domain-containing adapter molecule-1 (TICAM1), and LPS-binding protein (LBP)] show a spatial expression pattern in the epididymis, adding further credence to region-specific inflammatory responses in the epididymis (14, 15, 61, 63, 64).

The first demonstration that TLR4 activation by LPS was sufficient to elicit epididymitis came from a rat model of experimentally induced systemic endotoxemia (61). Intravenous LPS (0.1–1.0 mg/kg) injection triggered rapid inflammatory responses in the epididymis that were mediated by TLR4-dependent NF-kappa B activation and an upregulation of inflammatory mediators [i.e. *Il1b*, *Tnf*, *Nos2*, *Bdkrb1* (61, 65)]. Intraperitoneal LPS (3.0 mg/kg) injection in mice results in a similar upregulation of pro-inflammatory cytokines and induces morphological alterations (immune cell infiltrates, fibrosis) that are mostly restricted to the cauda epididymis (66). This response is absent from *Tnf^−/−^* mice and wild-type mice treated with the TNFα inhibitor pomalidomide, indicating that the induced inflammatory response is the primary source of tissue damage (66).

Similar to the effects seen in the UPEC mouse model, retrograde intravasal LPS injection in rodents resulted in a severe inflammatory reaction within the cauda (66, 67). These responses were more robust than those generated by systemic LPS administration (66). Intriguingly, when LPS was directly injected into the mesenchyme of the caput epididymidis, this region was locally responsive as indicated by an upregulation of several members of interleukin, NF-kappa B, TNF families, and downregulation of β-defensins (68, 69).

The data from these studies point to the involvement of TLR4 signaling in mediating the immunopathological changes seen in acute bacterial epididymitis. However, the exact mechanism governing these events—such as the contributions of damage-associated molecular patterns (e.g. S100A) as enhancers (70), different TLR4-dependent signaling pathways (MYD88, TICAM), and downstream inflammatory mediators of epididymitis progression and severity—have only just begun to emerge.

## Conclusions

The spatial differences in the structure and cellular composition of the epididymal epithelium and the region-specific gene expression patterns associated with sperm maturation are well known. Now, evidence is also accumulating to support a differential immune response throughout the epididymis. Most models point to a stronger inflammatory response to the same infectious or inflammatory challenges in the cauda compared to the caput epididymidis. The elevated immune response in the cauda prompts immunopathological alterations that can impair fertility. Although the caput is principally immunoreactive and the constituting cells can respond to inflammatory stimuli, the magnitude is at a much lesser extent. The data thus far have illustrated that strategically positioned MP subpopulations as well as region-specific expression of diverse immunomodulatory factors enable a precisely tuned balance between tolerance and defense at the opposing ends of the very same duct. Finally, the BEB seems to confer protection throughout the length of the epididymal duct, particularly from autoimmunity to sperm neoantigens. Going forward, research is necessary to better understand how this organ is able to maintain tolerance to autoimmunogenic spermatozoa at one end of the duct and ensure protection from ascending microbes at the other end. Then, work is warranted to delineate the functions of leukocytes in the steady-state epididymis, which currently remain an enigma.

## Author Contributions

CP, ES, AP, SB, and AM performed literature research and wrote the manuscript. All authors contributed to the article and approved the submitted version.

## Funding

This work was supported by a GRK 1871/2 International Research Training Group Giessen-Monash grant on “Molecular pathogenesis of male reproductive disorders” (AM, CP, AP, and SB) and a project grant BH 93/1-4 (awarded to SB) from the Deutsche Forschungsgemeinschaft.

## Conflict of Interest

The authors declare that the research was conducted in the absence of any commercial or financial relationships that could be construed as a potential conflict of interest.
